# Dataset of metaheuristics for the flow shop scheduling problem with maintenance activities integrated

**DOI:** 10.1016/j.dib.2021.106985

**Published:** 2021-03-22

**Authors:** Antonella Branda, Davide Castellano, Guido Guizzi, Valentina Popolo

**Affiliations:** Dipartimento di Ingegneria Chimica, dei Materiali e della Produzione Industriale, Università degli Studi di Napoli Federico II, Piazzale Tecchio, 80 80125 Napoli, Italy

**Keywords:** Scheduling, Flow shop, Preventive maintenance, Genetic algorithm, Harmony search

## Abstract

This data article presents a flow shop scheduling problem in which machines are not available during the whole planning horizon and the periods of unavailability are due to random faults. The experimental dataset consists of two problems with different sizes. In the largest one, about 2400 problems were analysed and compared with two diffuse metaheuristics: Genetic Algorithm (GA) and Harmony Search (HS). In the smallest, about 600 problems were analysed comparing the solution obtained with an exhaustive algorithm with those obtained by means of GA and HS. This dataset represents a test-bed for further works, allowing a comparison between the solution quality and the computation time obtained with different optimization methods. The substantial computational effort spent to generate the dataset undoubtedly represents a significant asset for the scientific community.

## Specifications Table

**Table:** 

Subject	Industrial Engineering
Specific subject area	Scheduling Flow shop systems with maintenance activities
Type of data	Matlab workspaces Table (Excel)
How data were acquired	Data were acquired by a generation of Flowshop Scheduling Problems, finding its solutions. Instruments: hardware, software Make and model and of the instruments used: Google Cloud Virtual Instance 72vCPU Intel Skylake and 270GB of memory.
Data format	Raw Analysed
Parameters for data collection	The parameters for data collection are: the number of machines in the flow shop system, the reliability and the number of jobs to be scheduled.
Description of data collection	Starting from a Design of Experiments, flow shop scheduling problems were generated considering different levels of each factor (parameter). The optimal solutions were calculated and collected for each problem.
Data source location	Institution: University of Naples Federico II City/Town/Region: Naples Country: Italy
Data accessibility	Public repository: Repository name: Mendeley Data Direct URL to data: http://dx.doi.org/10.17632/58X5fxx67y.1
Related research article	Branda, A., Castellano, D., Guizzi, G., Popolo, V., Metaheuristics for the flow shop scheduling problem with maintenance activities integrated, (2020) Computers & Industrial Engineering, ISSN 0360-8352, https://doi.org/10.1016/j.cie.2020.106989.

## Value of the Data

• The main purpose of this data is to provide a test bed of Flow Shop Scheduling Problem (FSSP) integrated with preventive maintenance and stochastic breakage. In particular, problems of different size and computational complexity were proposed in order to compare heuristic algorithms in solving problems similar to those in real industrial applications. The computational effort to solve such problems, finding the optimal sequence of jobs or a solution close to the optimal - depending on the size of the problem - represents an additional value of the data.
• In this research, it is proposed to separately solve two minimization problems: (i) makespan minimization; (ii) Earliness Tardiness Penalties (ETP) minimization; they have been resolved with the use of two diffuse metaheuristics for medium and large problems: Genetic Algorithm (GA) and Harmony Search (HS). Small problems are solved also using an exhaustive algorithm. This test bed and its solutions will benefit all researchers involved in the topic of scheduling in flowshop systems in order to compare the results obtained with other solving algorithms they have developed. New experiments could exploit the proposed results by comparing new heuristic algorithms. This comparison can be made both in terms of the quality of the solution (i.e. a solution with a better value according to one of the objective functions considered) and in terms of the time needed to compute the solution.

## 1 Data Description

To compare the performance of a Genetic Algorithm (GA) and Harmony Search (HS), two sets of scheduling problem are presented.

The first one deals with small problems. The proposed heuristics were compared with an exhaustive search method able to find the optimal solution for relatively small problems in a reasonable time. In this case, machines are more reliable (i.e., they require fewer maintenance activities resulting in a lower scheduling complexity).

Maintenance tasks are represented by Maintenance Jobs, the identification of the optimal sequence between job orders - that are 8, in small problems - and Maintenance Jobs - whose number depends on machine reliability (i.e., on the “beta” parameter) - on the different machines is the result of this dataset. Therefore, in each problem the optimal solution is proposed - for small-sized problems - or rather the best solution found with the two heuristics considered, for large-sized problems.

In the second set, which concerns more significant problems, the two heuristics were compared considering different scheduling scenarios created according to different optimization and problem generation criteria. In this case, the machines are less reliable (i.e., they are more likely to fail and require additional maintenance, resulting in a higher scheduling complexity).

For this class of problems, the dataset contains six classes of problems for each objective function (Makespan minimisation or ETP minimisation): three with low scheduling complexity and three with high scheduling complexity. For each class, 100 problems were solved for a total of 600 × 2 × 2 = 2400 experiments.

We solved the problems exhaustively on a Google Cloud virtual instance with the following features: 72vCPU Intel Skylake and 270GB of memory. The same was done for the two heuristic algorithms on a different virtual instance with the following features: 4vCPU Intel Skylake and 15 GB of memory.

The Stopping condition in both heuristic methods was set at 15 minutes of stall time (i.e., if the solution does not improve for 15 consecutive minutes, then the algorithm will stop).

The dataset can be downloaded from [1].

In the file “Problems.zip” 800 files named Problem1.mat, Problem2.mat ... Problem800.mat can be found (Table 1). Each file contains a MATLAB workspace with the following information:

• N is the number of Jobs.
• M is the number of Machines of the flowshop system.
• CL is the size of the problem (N+PM).
• s is the setup time matrix (the size is NxNxM): each cell (i,j,k) represents the setup time on machine k to switch from job i to job j. In the considered problems, it is assumed that the setup time to switch from job i to job j and the setup time to switch from job j to job i are the same.
• V is an array of size M. Each cell contains the time required for planned maintenance on each machine.
• R is an array of size M. Each cell contains the time required for corrective maintenance on each machine.
• Beta is an array of size M. Each cell contains the value of the shape parameter of the Weibull distribution on each machine.
• Eta is an array of size M. Each cell contains the value of the scale parameter of the Weibull distribution on each machine.
• TPO is the processing time matrix (the size is MxN): each cell (i,k) represents the processing time of the job i on the machine k.
• Z in an array of size M. Each cell contains the value of the number of the optimal planned maintenance on each machine.
• mu_DD is the mean of due date of the set of the J jobs.
• R_DD is the range of due date of the set of the J jobs.

**Table 1:** Description of the files in the folder “Problems.zip”.

Files named	Problem ID
“problem1.mat”, …, “problem100.mat”	SL1
“problem101.mat”, …, “problem200.mat”	SH1
“problem201.mat”, …, “problem300.mat”	ML1
“problem301.mat”, …, “problem400.mat”	LL1
“problem401.mat”, …, “problem500.mat”	LL2
“problem501.mat”, …, “problem600.mat”	MH1
“problem601.mat”, …, “problem700.mat”	LH1
“problem701.mat”, …, “problem800.mat”	LH2

The result of calculations is present in the file “Solutions.zip”.

Each folder (Table 2) contains three files: “solutions comparison.xlsx”, “GA solutions.xls” and “HS solutions.xls”. In folders “problems (001-100)” and “problems(101-200)”, the file “EA solutions.xls” is present.

**Table 2:** Description of the files in the folder “Solutions.zip”.

Folder named	Solution	Objective function
“problems(001-100)” “problems(101-200)”	contains the results and the comparison between the exhaustive method, and the two meta-heuristics	minimizes makespan
“problems(201-300) duedate” … “problems(701-800) duedate”	contains the comparison between the results of the two mete-heuristics	minimizes ETP
“problems(201-300) makespan” ... “problems(701-800) makespan”	contains the comparison between the results of the two meta-heuristics	minimizes makespan

The content of the Matlab file workspace (i.e. problem1.mat) can be viewed and exploited using open software such as python.

The following is an example script that uses the scipy library to load the workspace of a .mat file into python (release 3.x):

import scipy.io as sio
*'the variable mat_contents contains the workspace of the matlab file'*
*'assigning to the variable filename the matlab file with its path if it is different from the current directory'*
filename=*'problem1.mat'*
*'Load the .mat file contents.'*
mat_contents = sio.loadmat(filename)
*'The result is a dictionary, one key/value pair for each variable:'*
print (mat_contents.keys())
*'if you want to see the content of a variable of the workspace i.e. variable CL you have to write'*
print (mat_contents[*'CL'*])

## 2 Experimental Design, Materials and Methods

Problems are generated using the following rules used by [2]:

• Job processing time is a Gaussian random variable with mean 100 and standard deviation 25.
• Setup times are uniformly distributed between 0 and 19.
• Weibull eta (η) is 100.
• The average time required to carry out corrective maintenance is evenly distributed between 15 and 25.
• The average time required to carry out the planned maintenance is evenly distributed between 30 and 50.

For the problems related to the minimisation of ETP, these additional parameters are defined:

• Earliness penalty (a): equal to 1.
• Lateness penalty (b): equal to 8.
• Tardiness factor (TF): uniformly distributed between 0.3 and 0.6.
• Relative two date range (RD): equal to 0.4.

For the generation of due dates in a scheduling problem on a single machine the rule proposed by [3] was modified to be adapted to the scheduling problem with multiple machines:

$$\mu_{DD}=\frac{p}{N}*M*N*\left(1-TF\right)$$

$$R_{DD}=\frac{p}{N}*M*N*\text{RD}$$

Due dates are uniformly distributed with average µ_DD_ and range R_DD_.

Hence, the value of these parameters in individual problems are deterministic. To create different problems (each relating to machines and jobs characterized by different parameters), the aforementioned random procedures were adopted. Therefore, this dataset was generated using variables and parameters that cannot be found elsewhere.

The data, reported here, were used in [4] in order to schedule together production and maintenance activities in a flow-shop environment.

## Ethics Statement

It is not applicable.

## CRediT Author Statement

**Antonella Branda:** Software, Investigation, Validation; **Davide Castellano:** Writing - review & editing, Supervision, Project administration; **Guido Guizzi:** Software, Supervision, Resources, Writing - review & editing, Project administration; **Valentina Popolo:** Writing - original draft, Data curation, Visualization.

## Declaration of Competing Interest

The authors declare that they have no known competing financial interests or personal relationships which have or could be perceived to have influenced the work reported in this article.
